# Downregulation of MTAP promotes Tumor Growth and Metastasis by regulating ODC Activity in Breast Cancer

**DOI:** 10.7150/ijbs.67149

**Published:** 2022-04-24

**Authors:** Ying Zhang, Tian-Tian Zhang, Lin Gao, Ya-Nan Tan, Yu-Ting Li, Xiang-Yu Tan, Tu-Xiong Huang, Hua-Hui Li, Feng Bai, Chang Zou, Xin-Hai Pei, Bin-Bin Tan, Li Fu

**Affiliations:** 1 Guangdong Provincial Key Laboratory of Regional Immunity and Diseases, Department of Pharmacology and International Cancer Center, Shenzhen University Health Science Center, Shenzhen 518060, Guangdong, China.; 2 Shenzhen People's Hospital (The Second Clinical Medical College, Jinan University; The First Affiliated Hospital, Southern University of Science and Technology), Shenzhen 518020, Guangdong, China.; 3 Department of Clinical Oncology, The University of Hong Kong-Shenzhen Hospital, Shenzhen 518053, China.; 4 Department of Pathology, Shenzhen University Health Science Center, Shenzhen 518060, Guangdong, China.; 5 Department of Anatomy and Histology, Shenzhen University Health Science Center, Shenzhen 518060, Guangdong, China.

**Keywords:** ODC, MTAP, Polyamine biosynthesis, Breast cancer, Metastasis

## Abstract

5'-Methylthioadenosine phosphorylase (MTAP) is a key enzyme in the methionine salvage pathway and has been reported to suppress tumorigenesis. The MTAP gene is located at 9p21, a chromosome region often deleted in breast cancer (BC). However, the clinical and biological significance of MTAP in BC is still unclear. Here, we reported that MTAP was frequently downregulated in 41% (35/85) of primary BCs and 89% (8/9) of BC cell lines. Low expression of MTAP was significantly correlated with a poor survival of BC patients (P=0.0334). Functional studies showed that MTAP was able to suppress both *in vitro* and *in vivo* tumorigenic ability of BC cells, including migration, invasion, angiogenesis, tumor growth and metastasis in nude mice with orthotopic xenograft tumor of BC. Mechanistically, we found that downregulation of MTAP could increase the polyamine levels by activating ornithine decarboxylase (ODC). By treating the MTAP-repressing BC cells with specific ODC inhibitor Difluoromethylornithine (DFMO) or treating the MTAP-overexpressing BC cells with additional putrescine, metastasis-promoting or -suppressing phenotype of these MTAP-manipulated cells was significantly reversed, respectively. Taken together, our data suggested that MTAP has a critical metastasis-suppressive role by tightly regulating ODC activity in BC cells, which may serve as a prominent novel therapeutic target for advanced breast cancer treatment.

## Introduction

Breast cancer (BC) is the most commonly diagnosed malignancy worldwide and a leading cause of cancer-related deaths in women 1. The overall survival and prognosis have been substantially improved in recent years with the improvement of diagnosis and treatment, but metastases still lead to treatment failure and the molecular mechanisms responsible are poorly understood 1-4. Therefore, investigation of relevant mechanisms underlying BC invasion and metastasis may lead to identification of new prognostic markers and potential therapeutic targets.

5'-Methylthioadenosine phosphorylase (MTAP) is a key enzyme in the catabolism of 5'-Deoxy-5'-Methylthioadenosine (MTA) 5, 6 and has been reported to serve as a tumor suppressor gene 7-9. The MTAP gene is located at 9p21, a chromosomal region often deleted in BC, indicating that MTAP may also play an essential role in BC tumorigenesis. MTAP is ubiquitously expressed in normal cells and tightly regulates their DNA/protein synthesis and energy production 10. However, the expression of MTAP is frequently reduced or absent in various human cancers, including leukemia 11, lymphoma 12, lung cancer 13, pancreatic cancer 14, melanoma 15, 16 and myxofibrosarcoma 17. It has been reported that either homozygous deletion or promoter methylation may contribute to the downregulation of MTAP gene in human cancers 10, 18. Moreover, several studies have looked into the molecular mechanism underlying the tumor suppressor role of MTAP in human cancers. For example, downregulation of MTAP increases MTA, which is a selective inhibitor of the tumor-promoting gene PRMT5 (Protein arginine methyltransferase 5) 15, 19, 20. Additionally, downregulation of MTAP also promotes tumor metastasis by activating the GSK3β/Slug/E-cadherin axis in esophageal squamous cell carcinoma 21. MTAP cleaves MTA, a by-product of polyamine biosynthesis, into adenine and 5-methylthioribose-1-phosphate (MTR-1-P), which are further metabolized to methionine 22. Polyamines, including putrescine, spermidine, and spermine 23-25, are not only essential for a multitude of cellular physiological functions 26, 27, but also exert significant roles in tumor initiation and progression 28-32. To date, the effect of MTAP on the malignant behavior of BC and its potential regulatory role in polyamine biosynthesis pathway remains largely unclear.

In the present study, the expression pattern of MTAP in clinical BC samples and BC cell lines was studied. Both *in vitro* and *in vivo* assays were used to investigate the tumor suppressive function of MTAP. In addition, the possible mechanisms of MTAP in BC metastasis was also addressed.

## Materials and methods

### Tissue microarrays (TMAs) and immunohistochemistry (IHC)

Human breast cancer tissue microarray (TMA) slides with associated clinicopathological information were commercially available from Shanghai Outdo Biotech Co. Ltd. (Shanghai, China). Tissue specimens from BC patients with 85 tumor tissue and 55 adjacent non-tumor (NT) tissue samples were used in this study. Immunohistochemical (IHC) staining was performed to detect the expression of MTAP. The IHC staining assessment was independently conducted by 3 medical observers, one of whom is a pathologist. Comprehensive analysis was performed according to the staining intensity and the percentage of positive cells. The staining intensity was scored: 0 (unstained), 1 (light yellow), 2 (brown yellow), 3 (nigger-brown). The percentage of positive cell staining was graded as score: 0 (≤5%), 1 (6%~25%), 2 (26%~50%), 3 (51%~75%), 4 (76%~100%). The two scores were multiplied to be the positive grade: <2 (negative), ≥2 (weakly positive), ≥4 (moderately positive), ≥8 (strongly positive).

### Cell culture, reagents, and antibodies

All cell lines were purchased from the Cell Bank of Type Culture Collection of Chinese Academy of Sciences (Shanghai, China). These cell lines were identified by DNA-Fingerprinting, isozyme detection and mycoplasma detection. BT20, MCF-7, MDA-MB-231, MDA-MB-453, MDA-MB-468, HEK 293T, SW527, T47D were cultured in DMEM medium (Gibco, 11965092) plus 10% fetal bovine serum (FBS, Gibco, 10091155) and 1% penicillin/streptomycin (PS, Gibco, 15140163). BT474, BT549 were cultured in RPMI-1640 medium (Gibco, 12633012) plus 10% FBS and 1% PS. DFMO and Putrescine were purchased from Sigma-Aldrich. Antibodies used for western blotting were: Anti-β-actin (1:1000, 4970), Vimentin (1:1000, 5741) and Claudin-1 (1:1000, 13255) from CST, MTAP (1:1000, EPR6893) from Abcam. Antibodies used for IHC analysis were MTAP (1:100, EPR6893) and CD31 (1:100, GB11063-2) from Servicebio.

### Plasmids and shRNA

pReceiver-M12 and pEZ-M12 were purchased from GeneCopoeia, MTAP shRNA plasmids and control plasmid were purchased from Sigma-Aldrich. The shRNA sequence are as follows:

- shNTC: CCGGGATGACCAAGTGTGAGTGTAACTCGAGTTACACTCACACTTGGTCATCTTTTTG;
- shMTAP-1: CCGGGTCAACTACCAGGCGAACATCCTCGAGGATGTTCGCCTGGTAGTTGACTTTTTG;
- shMTAP-2: CCGGCAAGCCATCTGATGCCTTAATCTCGAGATTAAGGCATCAGATGGCTTGTTTTTG.

### Cell transfection, lentiviral packaging and infection

The plasmids were transfected into cells using the Lipofectamine® 3000 Reagent (L3000015, Thermo-Fisher Scientific, USA), then fresh medium was changed after 24 h. The lentiviral vector containing the inserted fragment and packaging vector (PAX and VSVG) were co-transfected into 293T cells to generate lentiviral particles. The cell supernatant was collected 48 h later, filtered by 0.45 μm filter membrane, and the virus was concentrated to infect the cells. Stably transfected cells were selected by puromycin (Thermo fisher, A1113803).

### RNA isolation and Quantitative Real-time PCR (qPCR)

Total RNA was extracted using Total RNA extraction kit (TaKaRa, 9767). Reverse transcription was performed using the PrimeScriptTM RT reagent Kit according to the manufacture's instruction (TaKaRa, RR047Q). Amplification by real-time PCR was performed using Bio-Rad CFX96^TM^ Real-Time PCR System (Bio-Rad, USA) according to the manufacturer's protocol. Cycle threshold (Ct) values were calculated, and the relative mRNA levels of targeted genes were analyzed using the ^2-ΔΔCt^ method. qPCR primer sequences were listed in Table S1.

### Western blot analysis

Total cellular proteins were extracted by the RIPA lysis buffer (Solarbio, China, R0010). The protein concentration was measured with BCA reagent (Thermo fisher, 23227). The proteins were separated by SDS-PAGE and then transferred to PVDF membrane (Thermo fisher, 22860). After blocking in 5% BSA for 1.5 h, the samples were incubated in suitable primary antibody at 4 ºC overnight and the secondary antibody at room temperature for 1.5 h. Immunoreactive bands were detected by enhanced chemiluminescence (ECL) reagent (Thermo fisher, A38555) and exposed using ChemiDoc MP imaging system (BioRad).

### Cell proliferation assay

Cells growth was quantified using the CCK-8 assay (Dojindo, CK04) according to the manufacturer's instructions. Briefly, cells were plated in 96-well plates (1×10^3^ cells/well) and incubated with 100 μl of medium overnight. The absorbance at 450 nm was measured using a microplate reader (Biotek, CYTATION 3) after culturing the cells with 10 μl of CCK-8 reagent. The P-values were carried out by GraphPad Prism with two-way ANOVA analysis.

### Migration and invasion assays

Cells were cultured in serum-free medium for 24 h before performing the migration and invasion assays. Cells in 0.5 ml of serum-free medium were seeded in the upper chamber (8 μm pore size, Corning, 353097) with or without 40 μl of 1 mg/ml Matrigel (BD, 356234), and 0.7 ml of complete medium containing 10% FBS was added to the lower chamber. After a period of incubation, the cells on the top of the membrane were removed with cotton swabs. Cells that had migrated/invaded to the bottom well were fixed with 4% paraformaldehyde for 10min and stained with a 0.5% crystal violet (Sigma-Aldrich, 548-62-9) solution for 15~30 min. The number of migrating/invading cells was counted in three randomly selected light microscopy fields (Carl Zeiss AxioVision AX10, magnification, 5×) by Image-J software.

### Tumor xenografts and IVIS Lumina Imaging

BT20-shNTC or BT20-shMTAP cells stably transfected with luciferase expression vector were injected into the mammary fat pads of 4-week-old female nude mice (n=5/group, 3×10^6^ cells per mouse). After one week, the primary tumor growth was monitored with a vernier caliper every two days, the tumor volume was determined by the formula: tumor volume = length × width^2^ × 1/2. The mice were sacrificed 24 days after transplantation. Tumors, lungs and lymph nodes of the mice were detected for bioluminescence imaging using an IVIS Lumina II imaging system (Perkinelmer, IVIS Spectrum, USA). The number of metastatic nodules in the lung was counted after fixation with 4% paraformaldehyde (PFA). Fresh tumor tissues, lung tissues and lymphoid tissues were fixed with 4% PFA and embedded in paraffin blocks for haematoxylin and eosin (H&E) staining and IHC staining. All animal experiments were approved by the Animal Care Committee of Shenzhen University Health Science Center.

### Tube formation assay

BT20-shMTAP and BT20-shNTC cells were cultured in serum-free medium for 48 h followed by conditioned medium (CM) collection. The matrigel and CM were fully mixed (50:50, v/v), and a 50 μl mixture was added into each well of a 96-well plate which was pre-chilled on ice and incubated for 1 h at 37 °C. HUVEC cells were seeded at a density of 2×10^4^ cells/well onto 96-well plate and incubated with 200 μl CM collected from the MTAP-knockdown BT20 cells and their control cells, respectively. After 3 h incubation, tube formation was observed and photographed under the microscope. Image analysis of tube formation was carried out using Image-J software.

### Metabolomic Experiments and LC-MS Analysis

Cells were seeded in 10 cm dishes and cultured to confluence. Cells were washed with PBS three times to thoroughly remove any remaining culture medium and ensuring that no PBS remains. Ice-cold 80%/20% (v/v) methanol/water (1 mL per 10 cm dish) was added into the plates and agitated to ensure that all cells were covered. Plates were scraped to dislodge cells, and lysates were transferred to an Eppendorf tube. The sample tubes were submerged into liquid N_2_ for 30s to sap freeze the cells and then allowed to thaw on dry ice. The freeze/thaw cycle was performed three times and the sample vortexed between each cycle. Lysates were centrifuged to pellet cell debris, and supernatants were transferred to a new Eppendorf tube. Samples were then dried under reduced pressure and stored at -80 °C until LC-MS analysis.

Extracted samples were analyzed by multiple LC-MS systems on a QExactive orbitrap mass spectrometer (Thermo Fisher Scientific, San Jose, CA). Briefly, an ACQUITY UPLC HSS T3 (1.8 μm, 2.1×100 mm) column (Waters, Eschborn, Germany) was used. The column oven was kept at 50 °C. LC separation was carried out using a mobile phase consisting of 0.1% acetic acid in water (Solvent A) and acetonitrile (Solvent B) at a flow rate of 350 µL/min. A Q Exactive Mass Spectrometer was used at 70,000 resolving power to acquire data in full-scan mode. Raw data were processed using XCMS software. Data analysis was performed by the business analysis software One-MAP (www.5omics.com). Resulting features were manually inspected for quality, and filtered for endogenously occurring metabolites.

### Statistical analysis

Statistical analysis was performed using GraphPad Prism software. Statistical comparison between the two groups was analyzed by student's *t*-test. Kaplan-Meier method and log-rank sum test were used to draw survival curves. Chi-square test was used to analyze the expression difference of MTAP in breast cancer and adjacent non-tumor tissues. P values < 0.05 were considered statistically significant.

## Results

### Low expression of MTAP is associated with poor prognosis in primary BC

**Figure 1:**
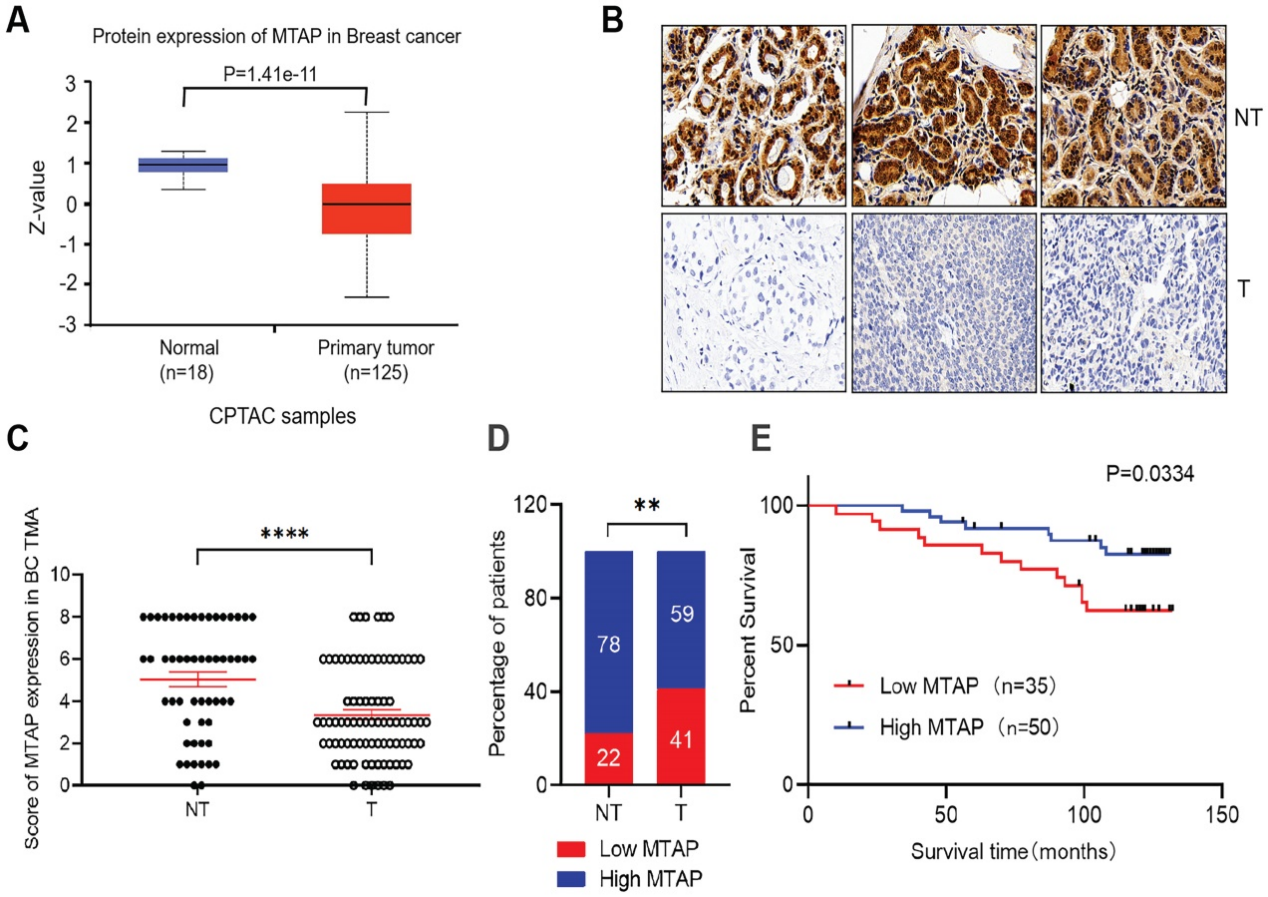
** Expression of MTAP and its prognostic significance in BC patients. (A)** Protein expression level of MTAP in normal and primary tumor tissues (CPTAC database). **(B)** Representative IHC staining showing the protein expression of MTAP in adjacent non-tumor (NT) and primary BC (T) from the TMA. Scale bar = 50 µm. **(C)** IHC scores of MTAP protein levels for 55 adjacent non-tumor (NT) and 85 primary BC (T) from the TMA. A score ≤ 2+ was defined as low expression of MTAP (Low MTAP), and score > 2+ was defined as high expression of MTAP (High MTAP). **** P<0.0001, student's *t*-test. **(D)** Histogram represents the percentage of MTAP expression status between 55 adjacent non-tumor (NT) and 85 primary BC (T) tissues. ** P<0.01, χ*2* test. **(E)** Kaplan-Meier Survival analysis according to MTAP expression in 85 primary BC patients (P=0.0334, log-rank test).

The protein levels of MTAP in primary BC and normal breast tissues were first analyzed using public database (http://ualcan.path.uab.edu/). In the clinical proteomic tumor analysis consortium (CPTAC) database, the levels of MTAP protein in primary tumor tissues (n=125) were significantly lower than that in normal breast tissues (n=18, P=1.41E-11, Figure 1A). To confirm the results drawn from the CPTAC database cohort, we detected the MTAP protein expression levels using TMA containing 85 primary BC and 55 adjacent NT tissues. The clinicopathological features of these cases were summarized in Table 1. Positive staining of MTAP was mainly observed at the cytoplasm (Figure 1B) and the expression of MTAP was classified into negative (scored as 0+), weak positive (scored as 2+), medium positive (scored as 4+) and strong positive (scored as 6+) staining. A score ≤ 2+ was defined as low MTAP and indicated as loss of expression for MTAP. A score > 2+ was defined as high MTAP and considered to have retained MTAP expression. The IHC scoring results confirmed that MTAP was significantly downregulated in primary BC compared with adjacent NT tissues (P<0.0001, Figure 1C). Moreover, low expression of MTAP was observed in 22% (12/55) of NT tissues and 41% (35/85) of primary BC, respectively, indicating that loss of MTAP expression is critical for the tumorigenesis of BC (P=0.0038, Figure 1D). The correlations of MTAP expression with various clinicopathological features were investigated and the results showed that low expression of MTAP was significantly positively associated with tumor recurrence (P=0.0261, Table 1). Furthermore, the result of log rank test showed that BC patients with low expression of MTAP (median survival time, 119 months) experienced a shorter overall survival (OS) than BC patients with high MTAP expression (median survival time, 125 months; P=0.0334, Figure 1E). These results suggested that loss of MTAP may play an important role in promoting the malignant progression of BC.

### MTAP suppresses the migration, invasion and EMT in human BC cells

**Figure 2:**
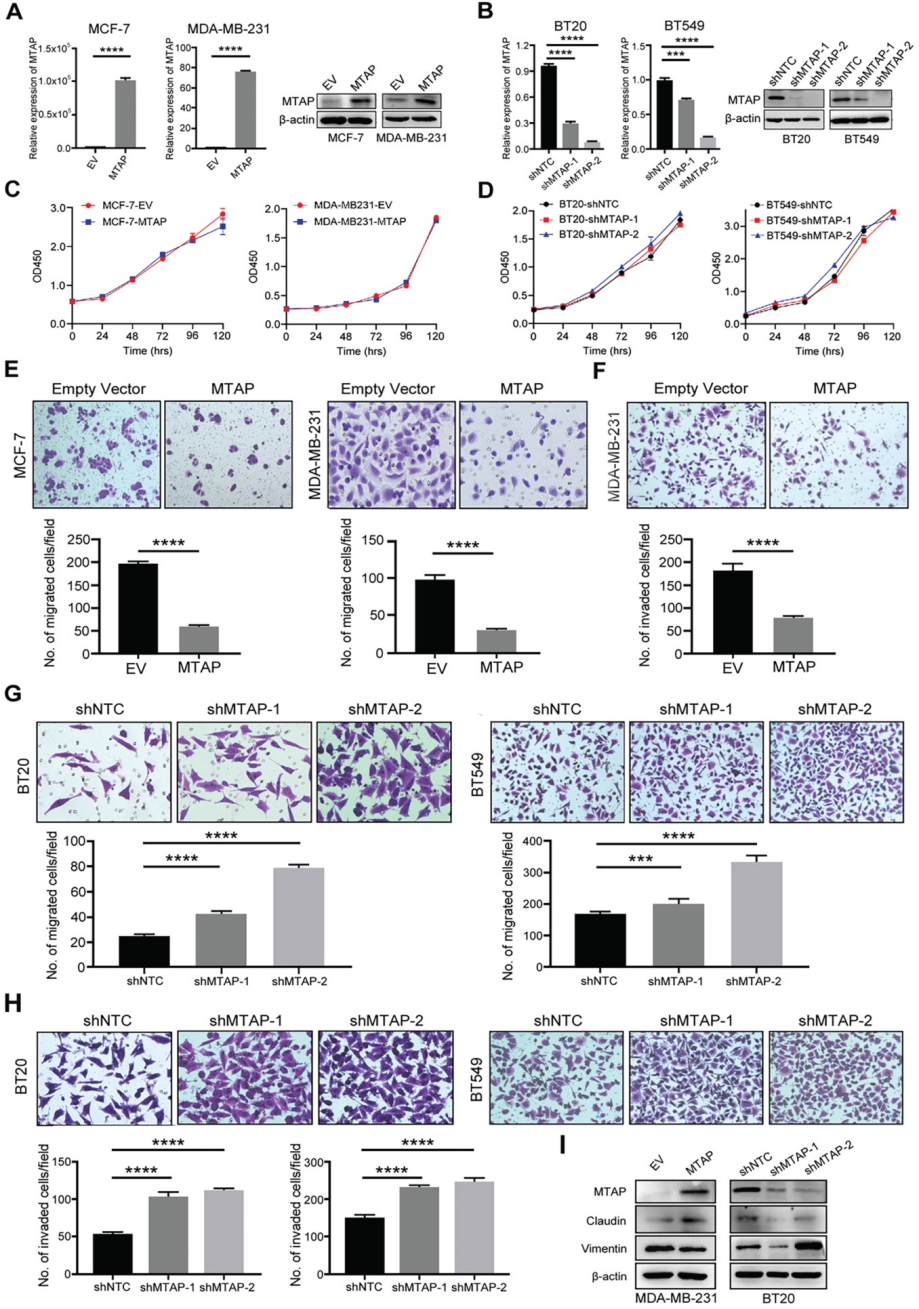
** MTAP regulates migration and invasion of BC cells *in vitro*. (A)** Overexpression of MTAP in MCF7 (MCF7-MTAP) or MDA-MB-231 (MDA-MB-231-MTAP) clones was confirmed by qPCR (left) and Western blotting (right). Empty-vector-transfected (MCF7/ MDA-MB-231-EV) cells were used as controls. **** P<0.0001, Student's *t*-test. **(B)** Knockdown of MTAP in BT20 (BT20-shMTAP-1/2) or BT549 (BT549-shMTAP-1/2) clones was confirmed by qPCR (left) and Western blotting (right). Non-template shRNA-transfected cells (BT20/ BT549-shNTC) were used as controls. *** P<0.001, **** P<0.0001, Student's *t*-test. **(C, D)** Cell viability was evaluated in MTAP-overexpressed (C) and -knockdown (D) BC clones every 24h for 5 days by CCK8 assay. The results are expressed as mean ± SD of three independent experiments. P > 0.05, Student's *t*-test. **(E)** Representative images of migration assays in MTAP overexpressed (MCF7/MDA-MB-231-MTAP) clones as compared to their controls. 1 × 10^5^ MCF-7 cells or 5 × 10^4^ MDA-MB-231 cells were allowed to migrate for 96 h or 24 h, respectively. Data are present as mean ± SD of triplicate experiments. **** P<0.0001, Student's *t*-test. **(F)** Representative images of invasion assays in MTAP overexpressed (MDA-MB-231-MTAP) clones as compared to their controls. 5 × 10^4^ MDA-MB-231 cells were allowed to invade into the Matrigel layer for 48 h. Data are presented as mean ± SD of triplicate experiments. **** P<0.0001, Student's *t*-test. **(G, H)** Representative images of migration (G) and invasion (H) assays of BT20/BT549-shMTAP-1/2 clones as compared to their controls. For migration assay, 6 × 10^4^ BT20 cells or 4 × 10^4^ BT549 cells were allowed to migrate for 26 h or 23 h, respectively. For invasion assay, 8 × 10^4^ BT20 cells or 7 × 10^4^ BT549 cells were allowed to invade into the Matrigel layer for 31 h or 27 h, respectively. Data are presented as mean ± SD of triplicate experiments. *** P<0.001, **** P<0.0001, Student's *t*-test. **(I)** Protein expression levels of EMT-related markers in MDA-MB-231-MTAP or BT-20-shMTAP-1/2 clones as compared to their controls.

To investigate the function role of MTAP in breast cancer cells, we initially examined the protein expression of MTAP in 9 BC cell lines (BT20, BT474, BT549, MCF-7, MDA-MB-231, MDA-MB-453, MDA-MB-468, SW527 and T47D) and a human breast epithelial cell line (Hs 578Bst) by Western blot (Figure S1). Absent or low expression of MTAP was detected in 8/9 (BT474, BT549, MCF-7, MDA-MB-231, MDA-MB-453, MDA-MB-468, SW527 and T47D) BC cell lines compared with the normal breast epithelial cell line Hs 578Bst. Next, we established stable MTAP-overexpressing clones in MCF-7 (MCF-7-MTAP) and MDA-MB-231 (MDA-MB-231-MTAP) cells without endogenous MTAP expression, respectively. Empty vector-transfected MCF-7 (MCF-7-EV) and MDA-MB-231 (MDA-MB-231-EV) cells were used as controls. MTAP mRNA and protein expression levels in MCF-7 and MDA-MB-231 cells were confirmed by qPCR and Western blot (Figure 2A). On the other hand, stable lines of BT20 and BT549 cells with lentivirus-mediated knockdown of MTAP expression (BT20/BT549-shMTAP-1/2) were also established. Non-template shRNA-transfected cells (BT20/BT549-shNTC) were established as controls. The knockdown efficiency of MTAP was confirmed using qPCR and Western blot (Figure 2B). The function of MTAP in BC cells was assessed by cell proliferation, migration and invasion assays. The cell counting kit-8 (CCK8) assays showed that either overexpression or knockdown of MTAP had no effect on the BC cell proliferation (Figure 2C, D). Compared with the control cells, MTAP overexpression dramatically inhibited cell migration of both MCF-7 and MDA-MB-231 cells (Figure 2E) and inhibited cell invasion of MDA-MB-231 cell (Figure 2F). As the invasion ability of control MCF-7 cells is relatively low, the data of its invasion suppression by MTAP was not shown. In contrast, MTAP knockdown promoted BT20 and BT549 cell migration (Figure 2G) and invasion (Figure 2H), respectively. Moreover, we detected the expression of EMT (epithelial-to-mesenchymal transition)-related markers by Western blot. The results confirmed that mesenchymal marker Vimentin was downregulated whereas epithelial marker Claudin was upregulated in the overexpressed BC cells compared with control cells (Figure 2I). In contrast, the expression of Claudin was decreased whereas the expression of Vimentin was increased in MTAP-knockdown BC cells compared with control cells (Figure 2I). These findings suggested that MTAP might play a critical role in the malignant progression of BC.

### Knockdown of MTAP increases tumor growth and metastasis of BC *in vivo*

**Figure 3:**
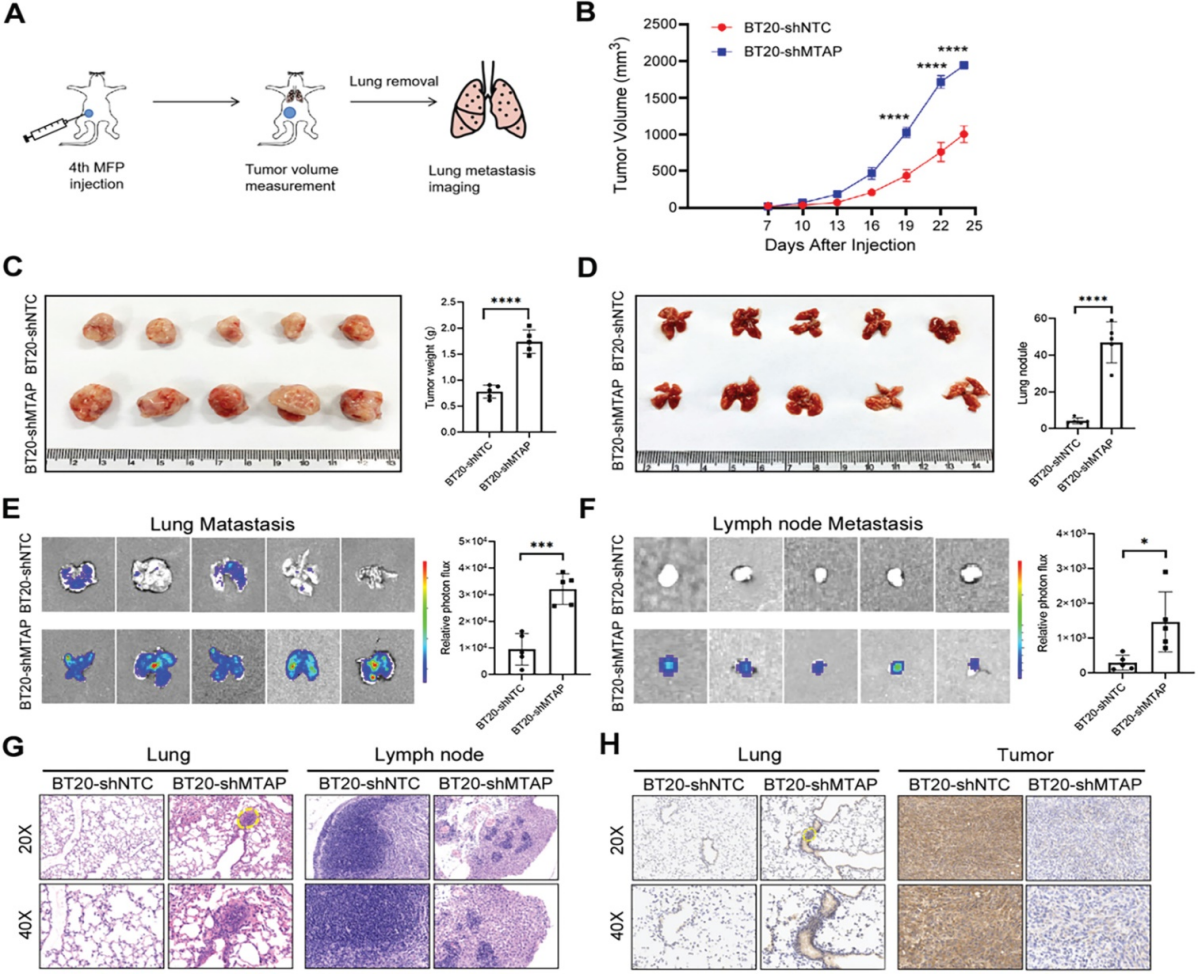
** MTAP knockdown promotes tumor growth and metastasis of BC *in vivo*. (A)** Schematic diagram showing the protocol of mouse model for human breast cancer spontaneous lung metastasis. **(B)** MTAP knockdown effectively promoted tumorigenicity in nude mice. n=5 per group, **** P < 0.0001, Two-Way ANOVA. **(C)** Representative image of the primary BC tumors removed from nude mice following injection of non-target control shRNA (shNTC) and MTAP shRNA (shMTAP)-transfected BT20 clones labeled with luciferase. The wet weight of primary tumors removed from BT20-shMTAP/Luc group or BT20-shNTC/Luc group was measured at 24 days post-injection. **** P < 0.0001, Student's *t*-test. **(D)** Representative image of lungs removed from BT20-shNTC/Luc group or BT20-shMTAP/Luc group at 24 days post-injection. Histogram representing mean ± SD of lung metastatic nodules from each group. **** P < 0.0001, Student's *t*-test. **(E)** *Ex vivo* bioluminescent images of lungs from BT20-shNTC/Luc group or BT20-shMTAP/Luc group. The area and intensity of bioluminescence represented the extent of lung metastasis. Plot representing mean ± SD of *ex vivo* lung photon flux. ***P < 0.001, Student's *t*-test. **(F)** *Ex vivo* bioluminescent images (left) and quantification (right) of lymph nodes metastasis from each group. Data are presented as the mean ± SD; * P < 0.05, Student's *t*-test. **(G)** Representative H&E staining images of lungs (left) and lymph nodes (right) from mice inoculated with BT20-shNTC/Luc or BT20-shMTAP/Luc. The marked portion represents the metastatic foci. Scale bar=50 µm. **(H)** Representative IHC staining images showing the expression of MTAP in lungs (left) and tumors (right) from mice inoculated with BT20-shNTC/Luc or BT20-shMTAP/Luc. Scale bar = 50 µm.

To explore the influence of MTAP on tumor growth and metastasis *in vivo*, a spontaneous BC metastasis model was established (Figure 3A). Firstly, we injected equal amounts (3×10^6^ cells) of luciferase-labelled BT20-shMTAP and BT20-shNTC cells into the fourth mammary fat pad of female BALB/c nude mice at 4 weeks old (n=5 per group). Due to the rapid growth of primary BT20 tumors, the volume was monitored by vernier caliper every 3 days. We then sacrificed the mice at 24 days post-injection to investigate whether MTAP was involved in the tumor growth and metastasis. Our results showed that the primary tumors in MTAP-knockdown group (BT20-shMTAP) grew faster than the control group (Figure 3B). The tumor volume caused by BT20-shMTAP cells (19.38±0.41 mm^3^) was significantly larger than tumors (10.03±1.15 mm^3^) induced by control cells (P<0.0001, Figure 3C). Moreover, the wet weight of tumors from BT20-shMTAP injected nude mice was significantly heavier than those from control group (P<0.0001, Figure 3C). These results indicated that knockdown of MTAP could effectively promote tumor growth *in vivo*. Furthermore, we removed the lungs and lymph nodes to monitor the metastasis through bioluminescent signal. The mice bearing MTAP-knockdown tumors had obviously higher nodules (Figure 3D) and bioluminescence (Figure 3E) in the lungs, as well as higher bioluminescence in the lymph nodes (Figure 3F). We further examined the metastatic foci in the lungs and lymph nodes by both H&E and IHC staining. As compared to the control group, H&E results showed heavy tumor burden (Figure 3G) while IHC results showed stronger MTAP staining (Figure 3H) in both lungs and tumors of the MTAP-knockdown group. Taken together, these results suggested that downregulation of MTAP promoted the tumor progression and metastasis in BC.

### Knockdown of MTAP promotes tumor angiogenesis in BC

**Figure 4:**
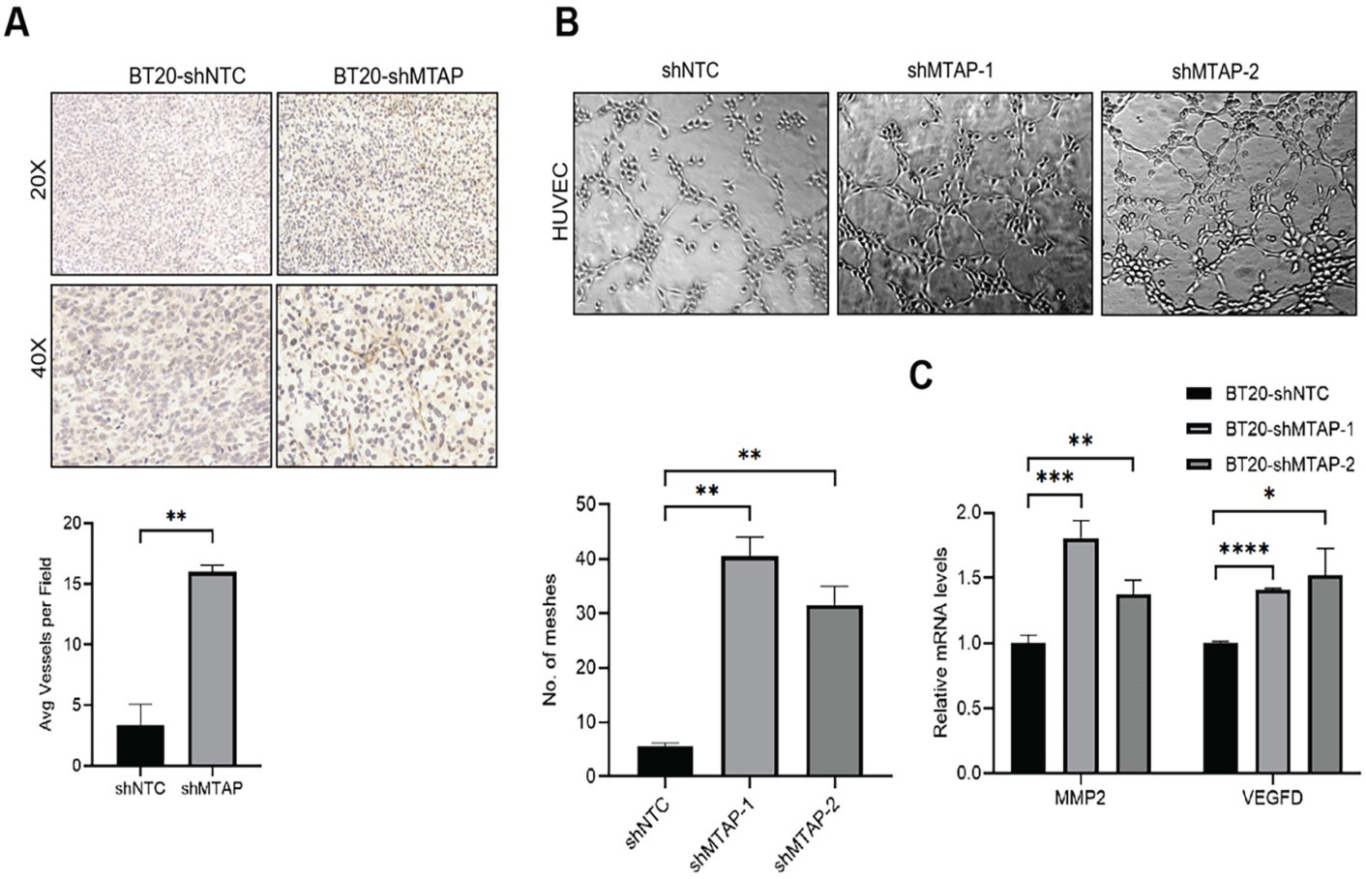
** MTAP knockdown promotes angiogenesis in BC. (A)** Representative IHC staining images showing the expression of angiogenesis marker CD31 in tumors from mice inoculated with BT20-shNTC/Luc and BT20-shMTAP/Luc clones. Scale bar=50 µm. The number of vessels in each field were quantified. ** P < 0.01, Student's *t*-test. **(B)** Representative images of HUVEC tube formation assay in of BT20-shMTAP-1/2 clones. BT20-shNTC was used as controls. The number of meshes were calculated. ** P < 0.01, Student's *t*-test. **(C)** mRNA expression of the MMP2 and VEGFD was evaluated by qPCR in MTAP-knockdown BT20 cells as compared to their controls. * P < 0.05, ** P < 0.01, *** P < 0.001, **** P < 0.0001, Student's *t*-test.

Notably, we found that primary breast tumors from MTAP knockdown group showed typical curvilinear tumor vessels, and hence we detected the angiogenesis in tumor tissues by CD31 immunostaining. As anticipated, vascular density quantification revealed that MTAP knockdown could induce more tumor vessels (Figure 4A). Next, we explored the effect of MTAP on tube formation of HUVECs and found that treatment with conditioned medium (CM) from MTAP-knockdown BT20 cells significantly enhanced the tube-forming ability of endothelial cells compared with their control cells (Figure 4B). To further investigate the potential mechanism underlying the angiogenesis-suppressing role of MTAP in BC, we initially queried GEPIA database to select 10 angiogenic genes showing significant and negative correlation with MTAP expression followed by qPCR validation. The results showed that only the mRNA expression levels of MMP2 and VEGFD were significantly upregulated in MTAP-knockdown BT20 cells (Figure 4C), while no such relationship was found in the remaining eight genes (Figure S2). Collectively, these findings suggested that MTAP may inhibit angiogenesis via downregulation of MMP2 and VEGFD expression.

### MTAP regulates amino acid metabolism by inhibiting polyamine biosynthesis in BC cells

To determine the effect of MTAP on the metabolism of BC cells, we compared the metabolic profiling between MDA-MB-231-MTAP and MDA-MB-231-EV cell extracts by using LC-MS. Six biological replicates were analyzed for each group. Normalization to the peak area sum was used as approximation for the average number of living cells which cannot be determined due to direct cell scraping. Relative metabolite concentration was normalized with the internal standard. A total of 142 annotated metabolites were detected by comparing the metabolite levels between these two groups. To analyze the metabolic pathways, the human metabolome database (HMDB) ID's of annotated metabolites were fed into the MetaboAnalyst database 33. Several metabolic pathways deregulated by MTAP were assigned, including cysteine and methionine metabolism, arginine and proline metabolism, and arginine biosynthesis (Figure S3). These results revealed that MTAP may play a critical role in regulating amino acid metabolism in BC cells.

**Figure 5:**
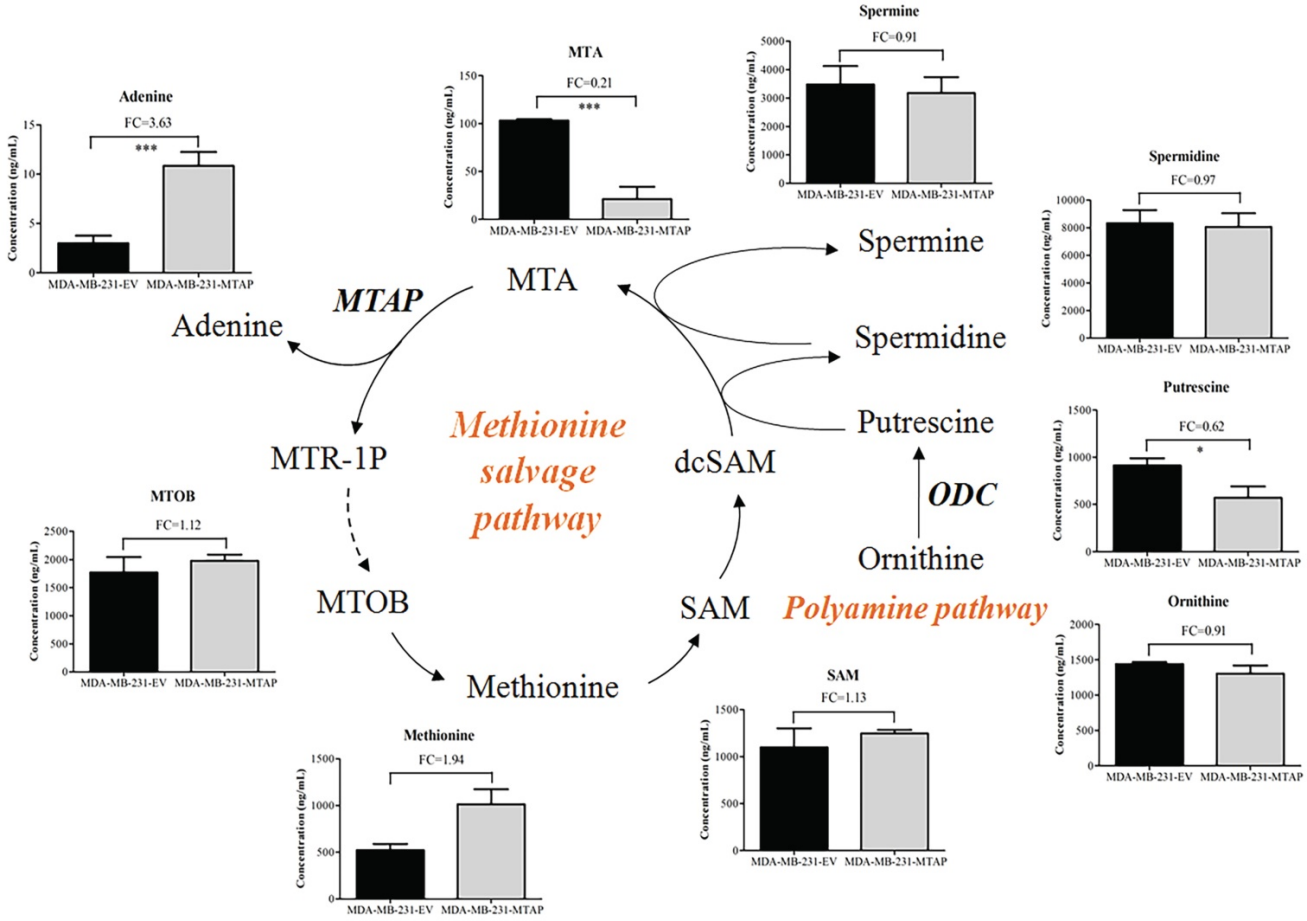
** Regulation of MTAP-related metabolic pathways by MTAP in BC cells.** Schematic diagram depicting two MTAP-related metabolic pathways (Methionine salvage pathway and Polyamine pathway). Each histogram next to the metabolite represents its relative levels detected in MTAP overexpressed MDA-MB-231 clones (MDA-MB-231-MTAP) as compared to their controls (MDA-MB-231-EV). MTA, 5'-Deoxy-5'-Methylthioadenosine; MTAP, methylthioadenosine phosphorylase; MTR-1P, 5-Methylthioribose-1-phosphate; MTOB, 4-methylthio-2-oxobutanoic acid; SAM, S-Adenosylmethionine; dcSAM, S-Adenosylmethioninamine; ODC, Ornithine Decarboxylase. * P < 0.05, ** P < 0.01, *** P < 0.001, Student's t-test. FC, Fold change was calculated by the average value of metabolite detected in MDA-MB-231-MTAP relative to their controls. FC > 1.0 indicates an increased level of metabolites while FC < 1.0 indicates a decreased level of metabolites in MDA-MB-231-MTAP vs MDA-MB-231-EV cells. Data are presented as mean ± SD of six independent experiments.

MTAP cleaves MTA into adenine and MTR-1P, which are further metabolized to methionine (Figure 5) 22. 4-Methylthio-2-oxobutanoic acid (MTOB) is the penultimate compound in the methionine salvage pathway which is used to recycle MTA. Methionine is converted to S-adenosylmethionine (SAM) which is the most popular methyl donor. SAM can also support polyamine synthesis when SAM is decarboxylated to form S-adenosylmethioninamine (dcSAM). Ornithine decarboxylase (ODC), the rate-limiting enzyme in polyamine biosynthesis, catalyzes ornithine to generate putrescine and subsequently spermidine and spermine. In these reactions, dcSAM is converted to MTA. Because MTAP is the only enzyme known to catalyze the degradation of MTA, we hypothesized that MTAP would affect the intracellular MTA level and related metabolic pathways. Based on the metabolic profiling analysis between MDA-MB-231-MTAP and MDA-MB-231-EV cell extracts, we comparative analyzed the following substances derived from the polyamine/methionine cycle: MTA, adenine, MTOB, methionine, SAM, ornithine, putrescine, spermidine, and spermine (Figure 5). Notably, MTA displayed the largest abundance decrease (FC=0.21, P<0.001), whereas Adenine, a byproduct of the methionine salvage pathway, displayed statistically significant increase (FC=3.63, P<0.001) in MTAP-overexpressing MDA-MB-231 cells compared with the control cells. Overexpression of MTAP in MDA-MB-231 cells also resulted in a modest increase (FC=1.12) in intracellular MTOB levels compared with the control cells. In contrast to MTA, the overall intracellular SAM levels showed no change based on MTAP status. Moreover, we found that all the polyamines (putrescine, spermidine, and spermine) levels demonstrated above showed a decreasing trend in MTAP-overexpressing MDA-MB-231 cells compared with the control cells. Specially, putrescine, the core product of ODC catalysis, displayed a statistically significant decrease (FC=0.62, P<0.05), suggesting that ODC activity might be inhibited by MTAP in BC cells.

### ODC is a crucial mediator of MTAP to regulate BC cell migration, invasion and angiogenesis

**Figure 6:**
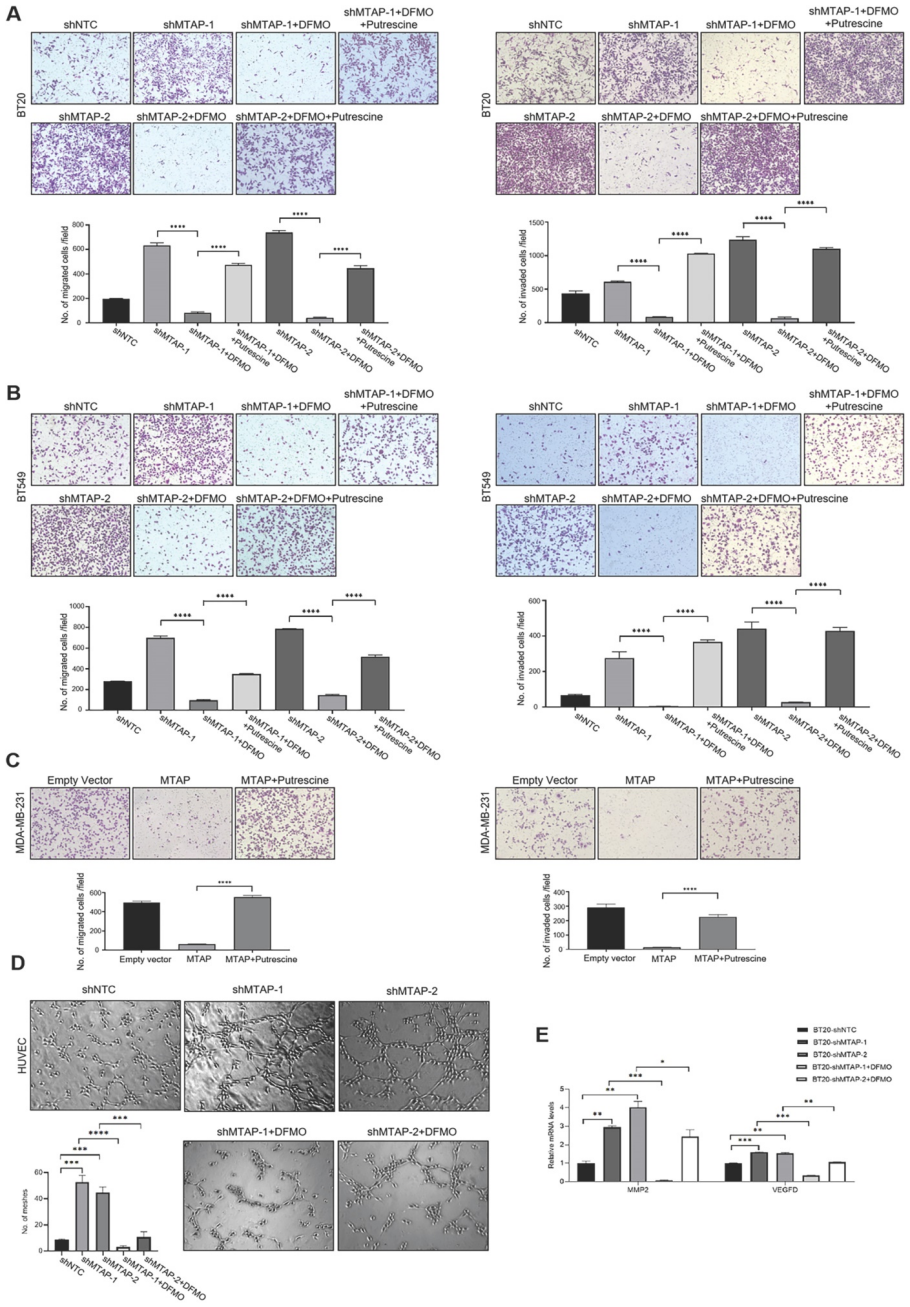
** The effects of exogenous putrescine and DFMO on MTAP-manipulated BC cells. (A)** Representative images of migration (left) and invasion (right) assays in MDA-MB-231-MTAP clones pretreated with normal culture medium containing 2 mM putrescine for 48 h. MDA-MB-231-MTAP clones without putrescine treatment or MDA-MB-231-EV cells were used as controls. Data are presented as mean ± SD of triplicate experiments. **** P < 0.0001, Student's t-test. **(B, C)** Representative images of migration (left) and invasion (right) assays in of BT20-shMTAP-1/2 (B) or BT549-shMTAP-1/2 (C) clones pretreated with normal culture medium containing 0.5 mM DFMO, or containing both 0.5 mM DFMO and 2.5 mM putrescine for 48 h. Data are presented as mean ± SD of triplicate experiments. **** P < 0.0001, as determined by Student's t-test. **(D)** Representative images of tube formation assays in HUVECs pretreated with conditional medium (CM) from BT20-shMTAP-1/2 clones containing 0 or 0.5 mM DFMO for 48 h. HUVECs pretreated with CM from BT20-shNTC cells were used as controls. The number of meshes of HUVECs in different treatment groups were calculated. *** P < 0.001, **** P < 0.0001, Student's *t*-test. **(E)** mRNA expression levels of the MMP2 and VEGFD were evaluated by qPCR in BT20-shMTAP-1/2 clones treated with 0 or 0.5 mM DFMO for 48 h as compared to their control cells. * P < 0.05, ** P < 0.01, *** P < 0.001, **** P < 0.0001, Student's *t*-test.

**Figure 7:**
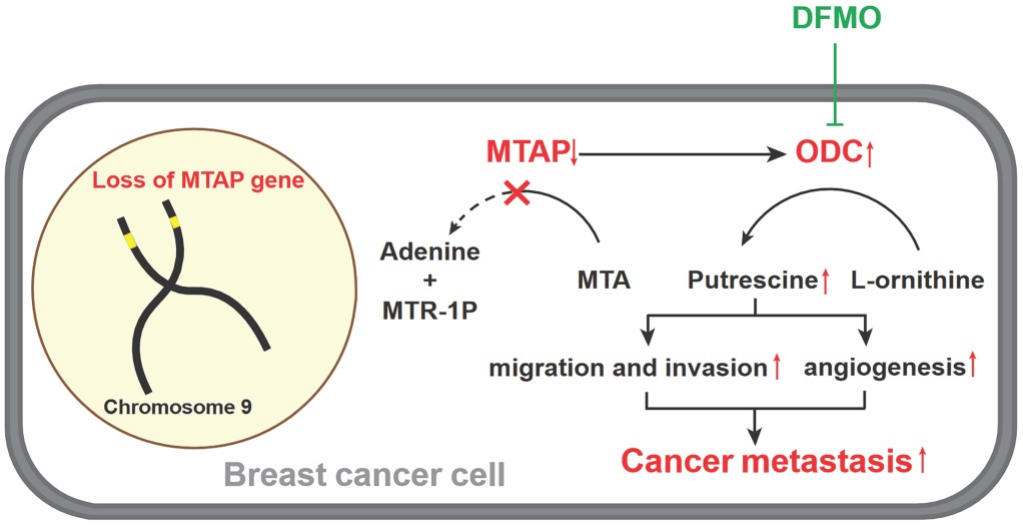
** A model for absent or low MTAP expression in promoting BC metastasis.** Downregulation of MTAP increases the polyamine levels by activating ODC, leading to BC metastasis. Treatment with specific ODC inhibitor DFMO reverses the metastasis-promoting phenotype of reduced MTAP in BC cells.

Based on the above metabolomics assay data, we speculated that MTAP may regulate the activity of ODC and therefore affect the synthesis of putrescine. In other words, overexpression or knockdown of MTAP directly regulate ODC activity which in turn regulate the synthesis of putrescine. To verify this conjecture, we treated BT20-shMTAP-1/2 and BT549-shMTAP-1/2 cells with DFMO, a specific inhibitor of ODC, and found the DFMO-treated cells significantly attenuated their migration and invasion capacity. Meanwhile, we wanted to determine whether the effect of DFMO on BC cell migration and invasion could be reversed by additional exogenous putrescine, which could further prove that ODC was acting through the polyamine pathway. As expected, the MTAP knockdown-enhanced migration and invasion capacities were inhibited by DFMO but restored by additional exogenous putrescine (Figure 6A, B). Moreover, the migration and invasion abilities in MDA-MB-231-MTAP cells were significantly up-regulated after additional exogenous putrescine, further supporting that putrescine has a strong metastasis-promoting role in BC (Figure 6C). In addition, to verify whether MTAP also played a role in tumor angiogenesis by regulating the ODC activity, we explored the effect of MTAP on tube formation of HUVECs and found that treatment with CM from MTAP-knockdown BT20 cells significantly enhanced the tube-forming ability of endothelial cells while additional DFMO in CM from MTAP-knockdown BT20 cells reversed the enhanced tube-forming ability of HUVECs (Figure 6D). Meanwhile, the mRNA expression levels of MMP2 and VEGFD were also significantly downregulated after DFMO treatment in MTAP-knockdown BT20 cells as compared to their control cells (Figure 6E). Collectively, these data indicated that MTAP could reduce the synthesis of putrescine through inhibiting the ODC activity, thereby suppressing BC metastasis (Figure 7).

## Discussion

Although the incidence of BC is on the rise globally, its prognosis has improved with the advancement of early diagnosis and treatment. At present, distant metastasis has the greatest impact on prognosis of BC. The BC patients without metastasis exhibit a five-year survival rate over 80% 3, while the five-year survival rate of patients with metastasis is only about 25% 4. However, to date the molecular mechanism of BC metastasis is not fully understood.

MTAP has been reported as a tumor suppressor gene in various types of human cancers, such as leukemia 11, lymphoma 34 and melanoma 16, 35, which is likely to be a potential target for cancer treatment. On the contrast, the exact role and related mechanism of MTAP in BC metastasis remains largely unclear. In this study, our TMA analysis confirmed that MTAP was abundantly expressed in non-tumor tissues but down-regulated in 41% (35/85) of BC tumor tissues. This result was consistent with the MTAP expression of BC in the CPTAC database. Previous studies have found that low MTAP expression was significantly associated with poor prognosis in non-small cell lung cancer 36, gastric cancer 37 and glioblastoma 38. Similarly, our study also showed for the first time that low expression of MTAP was positively correlated with tumor recurrence in BC patients, suggesting that MTAP may play an important role in the malignant progression of breast cancer.

In the present study, although MTAP overexpression could significantly inhibit BC cell migration and invasion while its knockdown had the opposite effect, an unexpected finding was that MTAP had no effect on BC cell proliferation *in vitro*. Interestingly, *in vivo* study showed that downregulation of MTAP could significantly promote both tumor growth and metastasis in BT20 cell-implanted orthotopic breast cancer model. The discrepancy between MTAP-mediated cell proliferating ability *in vitro* and *in vivo* indicated that MTAP may contribute to the BC tumor growth by regulating the tumor microenvironment. We indeed found that the primary BC tumors removed from the MTAP knockdown group had abundant tumor blood vessels compared with the control group. This observation was further confirmed by CD31 staining of the tumor sections from MTAP knockdown group and *in vitro* HUVEC tube formation assay. It has been reported that neovascularization plays an important role in the continuous growth of tumors and can provide them with more nutrients 39. Meanwhile, the abnormal morphology, such as incomplete endothelial lining and hyper-permeability of neovascularization, is also conducive to tumor invasion and metastasis 40. Therefore, it is possible that the influence of MTAP on angiogenesis also plays a significant role in BC tumor growth and metastasis.

Accumulating evidence suggests that MTAP can regulate tumor migration and invasion via different signaling pathways. Knockdown of MTAP can activate the GSK3β/Slug/E-cadherin axis and promote migration and invasion in esophageal cancer 21. Downregulation of MTAP can also modulate EMT and promote growth and metastasis in colorectal cancer 41. In melanoma, MTA accumulates when MTAP is downregulated, thereby promoting tumor metastasis through inhibiting protein methylation and activating ERK signal 15. Based on the cell metabolomics analysis, our present study found that MTAP overexpression significantly affected the levels of polyamine metabolites (especially putrescine) in BC cells. MTAP is the only metabolic enzyme that decomposes MTA into Adenine and MTR-1-P, and MTA is a by-product of polyamine biosynthesis 42. ODC is the rate-limiting enzyme in putrescine biosynthesis 43. It catalyzes the formation of putrescine from L-ornithine, which is the sole route to produce putrescine in mammals 11. In particular, ODC is considered as an independent prognostic factor of poor clinical outcome in breast cancer 44, 45. Numerous studies have reported that ODC and polyamine metabolism were closely associated with the growth, metastasis of tumor cells 29, 32, 46, 47; however, the toxicity of ODC inhibitor DFMO limits its clinical application 48, 49. Therefore, exploring the upstream regulatory pathways of ODC affecting tumor metastasis is promising to find new therapeutic targets. Previous studies in tissue samples from saccharomyces cerevisiae, pancreatic adenocarcinoma and neuroendocrine tumors showed that MTAP can inhibit ODC activity, thereby altering the polyamine pool and exerting a tumor suppressor effect 50, 51. In line with previous studies, our data showed that MTAP could also suppress the development and progression by regulating the ODC activity and putrescine level in BC cells. Moreover, we also found that MTAP may downregulate the expression of MMP2 and VEGFD in BC cells by inhibiting ODC activity, leading to the suppression of tumor angiogenesis. Although studies of MTAP in angiogenesis are scanty, a number of studies have demonstrated that ODC could regulate angiogenesis 52-54. In esophageal cancer 52 and breast cancer 53, overexpression of ODC was remarkably correlated with vascular invasion, lymph node metastasis and lymphovascular invasion. Meanwhile, several studies using animal models have found that treatment with the irreversible ODC inhibitor DFMO could inhibit tumor angiogenesis 55-57. However, there are only a few mechanistic studies illustrating how ODC regulates tumor angiogenesis. It has been reported that ODC participates in tumor invasion and metastasis by regulating MMP2 58. MMP2 can trigger the formation of tumor neovascularization 59. In our study, we also confirmed that the mRNA expression levels of MMP2 was significantly upregulated in MTAP-knockdown BT20 cells but downregulated after ODC inhibitor DFMO treatment, indicating MTAP could block breast tumor angiogenesis via inhibiting the ODC-MMP2 signaling axis. Collectively, overexpression of MTAP in BC cells could be an alternative approach to inhibit the ODC activity, which may provide a new strategy for individualized treatment of BC patients with metastasis.

In summary, our study demonstrated that MTAP may serve as a potential prognostic indicator for BC patients. Our study also elucidated that MTAP involves in the polyamine biosynthesis by regulating ODC activity, thereby suppressing breast tumor metastasis. These findings may provide insights to better understanding the polyamine biosynthesis pathway in the malignant progression of BC and lead to more effective management of individuals with MTAP deregulation.

## Supplementary Material

Supplementary figures and table.

## Figures and Tables

**Table 1:** Correlation between clinicopathological characteristics and MTAP expression in primary tumor tissue of 85 breast cancer patients

Clinicopathological features	Categorization	n	MTAP expression		P value^a^
			Low	High	
Age	≤ 54 years	43	21	22	0.1465
	> 54 years	42	14	28	
Tumor stage	TNM-I	19	7	12	0.901
	TNM-II	36	15	21	
	TNM-III	30	13	17	
Recurrence	Yes	21	13	8	**0.0261**
	No	64	22	42	

^a^, P value was calculated by using student's *t*-test; boldface indicates P value < 0.05.

## Abbreviations

MTAP: 5'-Methylthioadenosine phosphorylase
BC: Breast cancer
ODC: Ornithine decarboxylase
DFMO: Difluoromethylornithine
MTA: 5'-Deoxy-5'-Methylthioadenosine
PRMT5: Protein arginine methyltransferase 5
MTR-1-P: 5-methylthioribose-1-phosphate
TMA: Tissue microarray
IHC: Immunohistochemistry
NT: Non-tumor
CPTAC: Clinical proteomic tumor analysis consortium
OS: Overall survival
CCK8: Cell counting kit-8
EMT: Epithelial-to-mesenchymal transition
CM: Conditioned medium
GEPIA: Gene Expression Profiling Interactive Analysis
LC-MS: Liquid Chromatograph Mass Spectrometer
HMDB: Human metabolome database
MTOB: 4-Methylthio-2-oxobutanoic acid
SAM: S-adenosylmethionine
dcSAM: S-adenosylmethioninamine
