# Supplementary material for: Downregulation of MTAP promotes Tumor Growth and Metastasis by regulating ODC Activity in Breast Cancer
Source: Int J Biol Sci. 2022 Apr 24;18(7):3034–47. doi: 10.7150/ijbs.67149 (PMC9066107; doi:10.7150/ijbs.67149)
Supplement: Supplementary file 1 — Supplementary figures and table. [file ijbsv18p3034s1.pdf]

Downregulation Of MTAP Promotes Tumor Growth And Metastasis By Regulating ODC Activity In Breast Cancer

Ying Zhang[1], Tian-Tian Zhang[1], Lin Gao[2], Ya-Nan Tan[3], Yu-Ting Li[1], Xiang-Yu Tan[1], Tu-Xiong Huang[2], Hua-Hui Li[1], Feng Bai[4], Chang Zou[2], Xin-Hai Pei[5], Bin-Bin Tan[1*], Li Fu[1*]

Supplementary Tables

Table S1. qPCR primers

Primer	Sequence
human-ACTIN-F1	ACCTTCTACAATGAGCTGCG
human-ACTIN-R1	CCTGGATAGCAACGTACATGG
human-MTAP-F1	CAGGCGAACATCTGGGCTTT
human-MTAP-R1	GGACTGAGGTCTCATAGTGGT
human-TGFB1-F1	CTAATGGTGGAAACCCACAACG
human-TGFB1-R1	TATCGCCAGGAATTGTTGCTG
human-MMP2-F1	ACCCATTTACACCTACACCAAG
human-MMP2-R1	TGTTTGCAGATCTCAGGAGTG
human-MMP9-F1	CGAACTTTGACAGCGACAAG
human-MMP9-R1	CACTGAGGAATGATCTAAGCCC
human-VEGFB-F1	AGCCAGTGTGAATGCAGAC
human-VEGFB-R1	GTGGGATGGGTGATGTCAG
human-VEGFD-F1	TCCCATCGGTCCACTAGGTTT
human-VEGFD-R1	AGGGCTGCACTGAGTTCTTTG
human-ANG-F1	TGTTGGAAGAGATGGTGATGG
human-ANG-R1	CATAGTGCTGGGTCAGGAAG
human-ENG-F1	GCATCCTTCGTGGAGCTACC
human-ENG-R1	GAGGAGTGGTCTGGATCGG
human-PGF-F1	ACATGTTCAGCCCATCCTG

human-PGF-R1	TCCTTTCCGGCTTCATCTTC
human-TIMP1-F1	TTCTGCAATTCCGACCTCG
human-TIMP1-R1	TCATAACGCTGGTATAAGGTGG
human-TIMP4-F1	ATCTGTGCAACTACATCGAGC
human-TIMP4-R1	CGAGATGGTACAGGGTACTGTG

Supplementary Figures

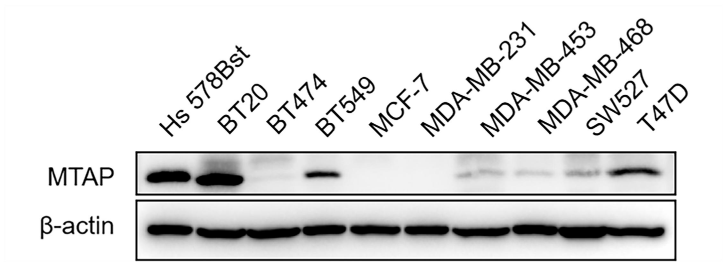

Figure S1. MTAP protein expression in BC cell lines and normal breast epithelial cell (Hs 578Bst) detected by Western blot. β-actin was used as a loading conctrol.

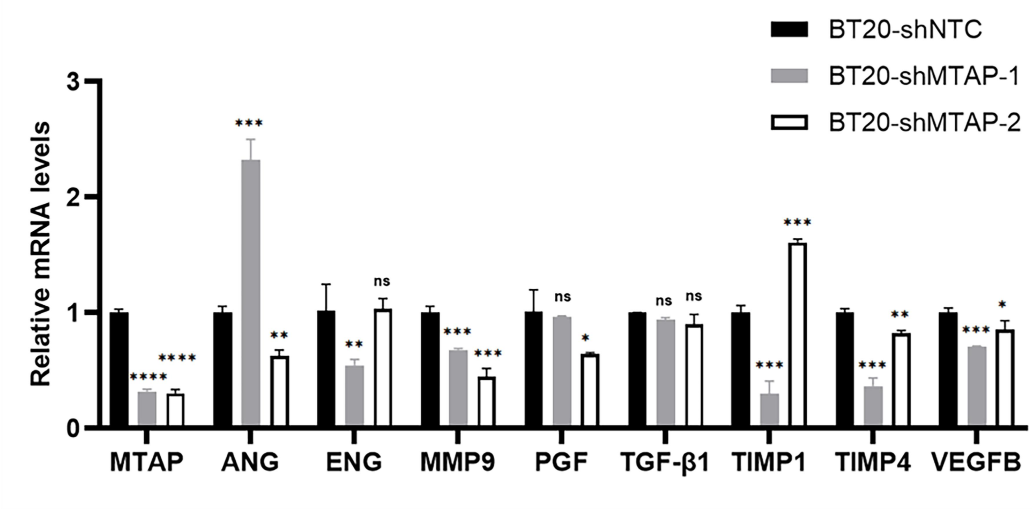

Figure S2. The expression of the 8 angiogenesis-associated genes was evaluated by qPCR in MTAP-transfected BT20 cells. ns, P > 0.05, * P < 0.05, ** P < 0.01, ***

P < 0.001,**** P < 0.0001, Student's t-test (above).

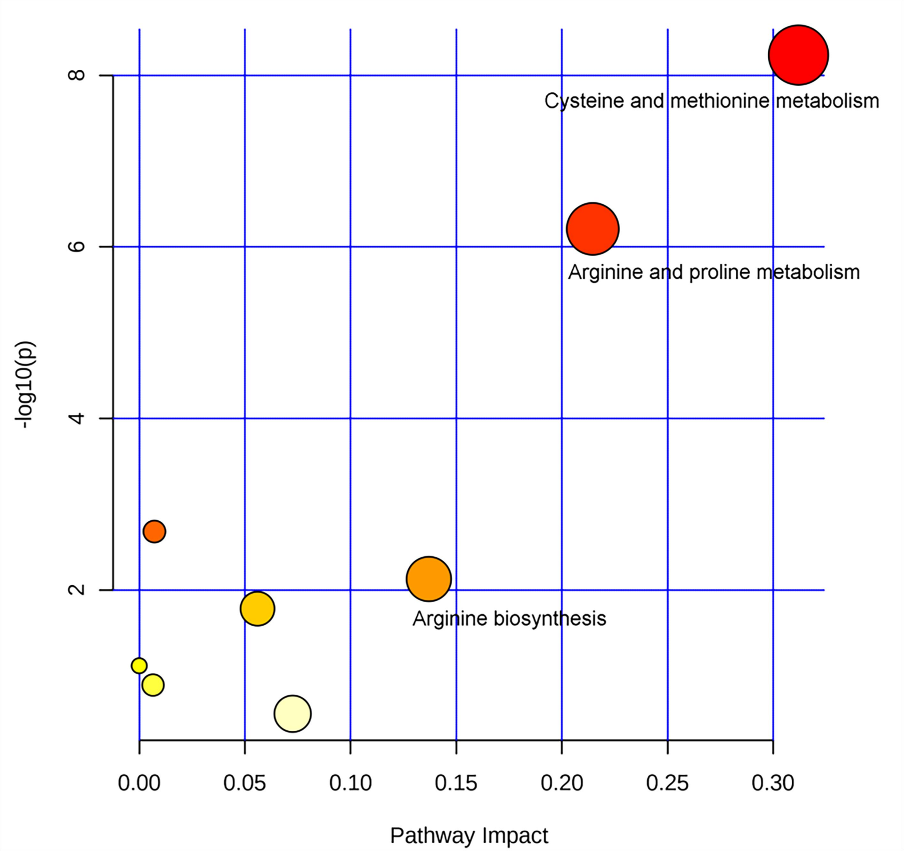

Figure S3. Summary of pathway analysis showing metabolic pathways arranged by scores from pathway enrichment and from topology analysis.
